# A Systematic Review and Meta-Analysis of Machine Perfusion vs. Static Cold Storage of Liver Allografts on Liver Transplantation Outcomes: The Future Direction of Graft Preservation

**DOI:** 10.3389/fmed.2020.00135

**Published:** 2020-05-12

**Authors:** Junjun Jia, Yu Nie, Jianhui Li, Haiyang Xie, Lin Zhou, Jun Yu, Shu-Sen Zheng

**Affiliations:** ^1^Division of Hepatobiliary and Pancreatic Surgery, Department of Surgery, First Affiliated Hospital, School of Medicine, Zhejiang University, College of Medicine, Hangzhou, China; ^2^Division of Hepatobiliary and Pancreatic Surgery, Department of Surgery, Key Laboratory of Combined Multi-organ Transplantation, Ministry of Public Health, Hangzhou, China

**Keywords:** machine perfusion, static cold storage, graft preservation, liver transplantation, meta-analysis

## Abstract

**Background:** Machine perfusion (MP) and static cold storage (CS) are two prevalent methods for liver allograft preservation. However, the preferred method remains controversial.

**Aim:** To conduct a meta-analysis on the impact of MP preservation on liver transplant outcome.

**Methods:** PubMed, EMBASE, and Cochrane Library databases were systematically searched to identify relevant trials comparing the efficacy of MP vs. CS. Odds ratios (OR) and fixed-effects models were calculated to compare the pooled data.

**Results:** Ten prospective cohort studies and two randomized controlled trials (RCTs) were included (MP livers vs. CS livers = 315:489). Machine perfusion demonstrated superior outcomes in posttransplantation aspartate aminotransferase levels compared to CS (*P* < 0.05). The overall incidence of early allograft dysfunction (EAD) was significantly reduced with MP preservation than CS [OR = 0.46; 95% confidence interval (CI) = 0.31–0.67; *P* < 0.0001]. The incidence of total biliary complications (OR = 0.53; 95% CI = 0.34–0.83; *P* = 0.006) and that of ischemic cholangiopathy (OR = 0.39; 95% CI = 0.18–0.85; *P* = 0.02) were significantly lower in recipients with MP preservation compared with CS preservation. Hypothermic machine perfusion (HMP) but not normothermic machine perfusion (NMP) was found to significantly protect grafts from total biliary complications and ischemic cholangiopathy (*P* < 0.05). However, no significant differences could be detected utilizing either HMP or NMP in primary nonfunction, hepatic artery thrombosis, postreperfusion syndrome, 1-year patient survival, or 1-year graft survival (*P* > 0.05).

**Conclusions:** Machine perfusion is superior to CS on improving short-term outcomes for human liver transplantation, with a less clear effect in the longer term. Hypothermic machine perfusion but not NMP conducted significantly protective effects on EAD and biliary complications. Further RCTs are warranted to confirm MP's superiority and applications.

## Introduction

Liver transplantation (LT) has been the optimal treatment for patients with end-stage liver disease. Rapidly increasing demands for LT have also caused severe shortage of donor liver organs in clinical application. To overcome this discrepancy, donation after circulatory death (DCD), extended criteria donors (ECDs), living liver donation, or marginal liver grafts are increasingly accepted to expand the limited donor pool. However, studies have suggested that these additional liver donation sources often suffered from longer warm ischemic periods and worse ischemia–reperfusion injury (IRI) during LT, resulting in increased risk of early allograft dysfunction (EAD), primary nonfunction (PNF), biliary complications, and poorer long-term graft and patient survival (1).

During transplantation, the quality of donor liver preservation is an important factor on maintaining and improving transplant outcomes of patients. Standard static cold storage (CS) remains the gold standard method for liver graft preservation. Nevertheless, the effectiveness of CS preservation is currently unable to provide sufficient protection of liver grafts against IRI, especially for prolonged CS of DCD and marginal livers, often resulting in increased risk of EAD, PNF, and biliary complications (2). As an alternative preservation strategy, machine perfusion (MP) can provide a continuous circulation of metabolic substrates and antioxidants, imitating the physiological processes while flushing inflammatory cytokine and toxins from the graft. Therefore, MP has been proposed as a better strategy to protect or rescue marginal liver grafts by attenuating the cytokine mediated IRI (3).

Machine perfusion has been widely used for preservation in kidney transplantation. Plenty of evidence suggested MP's priority in improving early graft function, reducing the risk of delayed graft function (DGF), and enhancing ECD graft survival after renal transplantation (4). Unlike kidney transplantation, MP remained relatively limited in clinical LTs due to the high metabolism rates of liver, relatively complicated perfusion system consisting of hepatic and portal route, poor practicality, and high costs.

Large animal and human experimental studies have suggested that MP may provide better protection of liver grafts against IRI than CS (5, 6). Expression of proinflammatory cytokines, oxidation markers, and activation of adhesion molecules and migration of leukocytes were significantly reduced by MP (7). Recently, two systematic review and meta-analyses concluded that MP preservation is superior to CS in experimental rat and pig models in terms of reducing hepatocellular injury [lower aspartate aminotransferase (AST), alanine transaminase (ALT), lactate dehydrogenase levels] and biliary injury (lower alkaline phosphatase, hyaluronic acid levels) (8, 9). Clinical adoption of MP in LT has been intensively tested. Since the first clinical series investigating the safety and efficacy of MP compared with CS was reported by Guarrera et al. (10), increasing comparative studies on MP and CS has been reported, showing the potential benefit in improving early graft function, reducing posttransplant complications, and enhancing long-term survival (11). However, those benefits on outcomes were inconclusive without the pooled analyses. Few systematic review and meta-analyses have tried to investigate the effectiveness of MP on human, but they were either short comprehensive or out-of-the-latest studies (12, 13). Updated systematic review and meta-analyses are lacking and needed to decide the preferred method of graft storage.

In this systematic review and meta-analysis, we systematically analyzed the available published data to compare MP preservation with CS preservation on outcomes of LT, including EAD, PNF, biliary complications, and graft and patient survival after transplantation, with the aim of identifying the optimum preservation method for donor livers and achieving preferable outcomes in clinical application.

## Methods

### Literature Search

A comprehensive literature search was carried out on PubMed/MEDLINE, EMBASE, and Cochrane library databases using the following terms: ((machine perfusion OR machine preservation) AND (liver OR liver transplant OR liver transplantation)). The literature search was performed in November 2019. Publications were limited to articles written in English. The titles and abstracts of all relevant publications were reviewed, and full texts of those potentially matching the inclusion criteria were retrieved. Reference lists of the retrieved full-text articles were also searched manually to help identify potentially eligible studies. This systematic review and meta-analysis was performed in line with the PRISMA (Preferred Reporting Items for Systematic Reviews and Meta-Analyses) recommendations (14).

### Study Inclusion and Exclusion Criteria

Clinical studies comparing outcomes of human LT using MP preservation vs. static CS were eligible for this analysis.

Exclusion criteria were as follows: (1) studies without a control group (without CS preservation group); (2) studies not controlling the donor type between the MP group and CS group; (3) studies using unusual MP method (including subnormothermic MP and controlled rewarming MP); (4) overlapping studies from the same institution; (5) studies reporting none of the following outcomes of interest: EAD, PNF, biliary complications, vascular complications, graft survival, and patient survival; (6) simultaneous liver–kidney transplantation, multiple organ transplantation, and living donor transplantation studies; (7) nonhuman studies and experimental studies; (8) abstracts, letters, editorials, books, expert opinions, case reports, and review articles.

### Quality Evaluation and Data Extraction

Newcastle–Ottawa Quality Assessment Scale (NOS) was used to evaluate the methodological quality of the included prospective cohort studies (15). The quality of randomized controlled trial (RCT) study was assessed using the Jadad score and the Cochrane Collaboration's risk of bias tool (16). Studies with score >6 were regarded as high quality. Two investigators (J.J. and Y.N.) reviewed the publications, assessed the quality, and extracted the data independently. Disagreements were resolved by discussion and consensus, confirmed by another investigator (S.-S.Z.).

The primary outcomes extracted included the incidence of EAD, PNF, hepatic artery thrombosis (HAT), total biliary complications, and ischemic cholangiopathy (IC) rates after transplantation. Secondary outcomes included liver graft function, postreperfusion syndrome (PRS), and 1-month, 6-month, and 1-year graft and patient survival rates.

All of the studies defined EAD according to Olthoff et al. (17). To be specific, EAD was defined as the presence of at least one of the following at 7 days after LT: serum bilirubin 10 mg/dL; or international normalized ratio 1.6; or ALT >2,000 U/L in the first 7 postoperative days. Primary nonfunction was indicated as irreversible graft dysfunction, for nontechnical and nonimmunological causes, leading to death or emergency liver replacement during the first 10 days after LT (18). Definition of IC was according to Lee et al. (19). Total biliary complications at 1 year were defined as all the complications related with biliary duct including biliary leaks, biliary stricture, and other biliary complications. Postreperfusion syndrome was defined according to Hilmi et al. (20).

### Statistical Analysis

Pooled odds ratios (ORs) were used to evaluate the summary event rates (dichotomous data) with 95% confidence interval (CI). Standardized mean difference (SMD) was used to compare continuous data. Cochran Q (χ^2^) and the *I*^2^ statistical test were adopted to evaluate the heterogeneity among the included studies. Heterogeneity was considered significant when *P* < 0.10 or *I*^2^ > 50%. In the absence of heterogeneity among the included studies, a fixed-effects model was adopted to combine studies and pool the total effect size; otherwise, a random-effects model was adopted. Funnel plots were created for assessment of publication bias (publication bias was examined in funnel plots by performing Begg and Egger tests). *P* < 0.05 was considered statistically significant. All statistical analyses were performed using Cochrane Review Manager 5.3. (http://ims.cochrane.org/revman).

## Results

### Search Results and Study Characteristics

**Figure 1:**
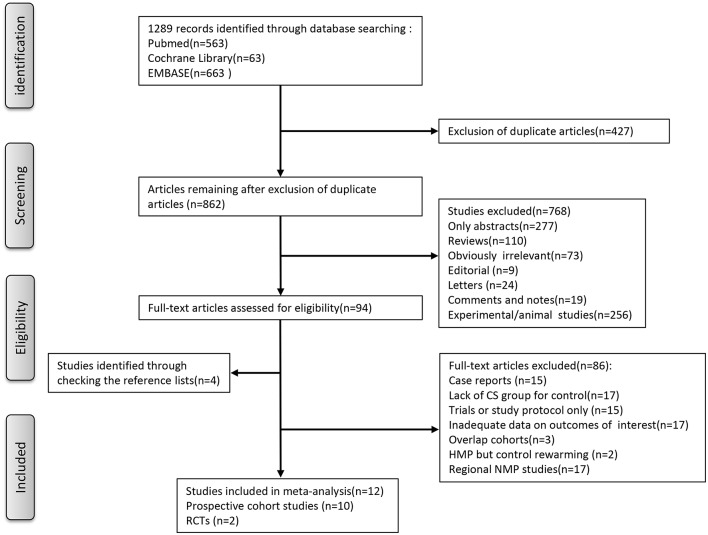
Flowchart showing search strategy with inclusions and exclusions.

As for the selection process according to the PRISMA guidelines, the detailed process of our literature search is shown in Figure 1. Briefly, Initial literature searches identified 1,289 publications. After excluding ineligible publications based on the selection and exclusion criteria, 12 studies, including 10 nonrandomized prospective phase I clinical trials (10, 21–29), one multi-institutional randomized study (18), and one single-center, randomized controlled study (30), were eventually included in the meta-analysis of the comparison between MP and CS on clinical outcomes in LT. In the 12 included studies, five studies used hypothermic machine perfusion (HMP), whereas the other seven studies investigated normothermic machine perfusion (NMP). Of these 12 studies, seven were from European institutes, including four single-center prospective trials, one multicenter prospective trials (22, 25–27), one prospective multicenter RCT (18), and one single-center, randomized controlled study (30); two single-center prospective trials were from Canadian institutes (23, 24), whereas the remaining three studies were from the US institutes (10, 21). The years of publication spanned from 2010 to 2019. After combining all the studies, 315 patients were included into the MP group, and 489 patients were included into the CS group as control in our meta-analysis.

The characteristics of each study are summarized in Table 1. The outcomes of each study are presented in Table 2 and Supplementary Data. As shown in Table S1, the quality of the 12 included studies was good. The quality scores of included cohort studies, evaluated by the NOS, ranged from 7 to 9 points (with a mean of 7.9), whereas the risk of bias in the two included RCTs was low.

**Table 1:** Clinical characteristics of the included studies in the meta-analysis.

**References**	**Institution**	**Study type**	**Study period**	**Donor type**	**MP Type**	**MP system**	**Sample Size**		**Donor age**		**Recipient age**		**Recipient meld scores**		**CIT hours**		WIT^*^ min		**Follow–up period**
							**MP**	**CS**	**MP**	**CS**	**MP**	**CS**	**MP**	**CS**	**MP**	**CS**	**MP**	**CS**	
Guarrera et al. (10)	USA	PCS	2004.7– 2008.2	DBD	HMP	Organ Assist	20	20	39.4 ± 2.5	45.6 ± 2.1	55.4 ± 6.2	52.7 ± 8.9	17.2 ± 7.4	16.8 ± 6.8	9.4 ± 2.1	8.9 ± 2.8	44.3 ± 6.5	45.1 ± 6.7	1 year
Dutkowski et al. (22)	U.K./ Europe MCT	PCS	2012.1– 2014.12	DCD	HMP	Organ Assist	25	50	54(36–63)	48(33–51)	60(57–64)	56(49–59)	13(9–15)	16(10–21)	3.1(2.4–4.4)	6.6(5.8–7.5)	36(31–40)	33(27–40)	1 year
Guarrera et al. (21)	USA	PCS	2007– 2012	ECD	HMP	Organ Assist	31	30	57.5 ± 17.8	57.9 ± 16.9	57.5 ± 8.0	58.4 ± 9.6	19.5 ± 5.9	21.4 ± 6.3	9.3 ± 1.6	8.6 ± 2.4	45.6 ± 7.3	40.0 ± 8.3	1 year
Ravikumar et al. (25)	UK	PCS	2011.1– 2013.12	DBD/ DCD	NMP	OrganOx metra	20	40	58.0(21–85)	58.5(21–82)	54.4(33–66)	55.0(27–65)	12(7–27)	14(6–25)	9.3(3.5–18.5)	8.9(4.2–11.4)	21(14–31)	15(9–23)	6 month
Selzner et al. (23)	Canada	PCS	2015.6– 2015.12	DCD	NMP	OrganOx metra	10	30	48(17–75)	46(22–68)	56(45–71)	54(42–63)	21(8–40)	23(7–37)	9.8(3.7–12.2)#	10.6(8.7–13.0)#	49(21–76)	46(39–67)	3 month
Bral et al. (24)	Canada	PCS	2015.2– 2015.12	DBD/ DCD	NMP	OrganOx metra	10	30	56(14–71)	52(20–77)	53(28–67)	59(43–69)	13(9–32)	19(7–34)	2.8(1.6–4.9)	3.9(1.1–14.8)	21.5(16–26)	N/A	6 month
van Rijn et al. (26)	The Netherlands	PCS	2014.4– 2014.11	DCD	HMP	Organ Assist	10	20	53(47–57)	53(47–58)	57(54–62)	52(42–60)	57(54–62)	52(42–60)	8.7(7.8–9.9)	8.4(7.9–8.8)	27(23–43)	32(27–39)	1 year
Watson et al. (27)	UK	PCS	N/A	ECD^**^	NMP	Organ Assist	12	24	56(24–67)	54(22–72)	57(46–65)	N/A	17(10–26)	N/A	7.1(3.7–14.6)	7.3(5.6–12.0)	31(17–160)	22(12–124)	1 year
Nasralla et al. (18)	U.K./ Europe MCT	RCT	2014.6– 2016.3	DBD/ DCD	NMP	OrganOx metra	121	101	56 (16–84)	56 (20–86)	55 (20–72)	55 (22–70)	13 (6–35)	14 (6–29)	2.2(0.8–3.6)	7.8(3.7–16.0)	21(9–93)&	16(2–32)	1 year
Ghinolfi et al. (30)	Italy	RCT	2016.10– 2018.4	ECD (DBD,≥ 70 years)	NMP	Organ Assist	10	10	90	80	57 (46–61)	55 (43–61)	12.5 (9–16)	9.5 (8–15)	4.7 (4.0–5.0)	6.6 (6.1–7.8)	74 (70–82)	69 (62–78)	1 year
Patrono et al. (29)	Italy	PCS	2016.03.16–2018.06.12	ECD	HMP	Organ Assist	25	50	74.3 ± 10.9	74.9 ± 10.3	56.3 ± 9	55.9 ± 7.4	15.3 ± 8.6	15.5 ± 8.5	5.2 ± 0.9	6.5 ± 1.2	23 ± 7	24 ± 5	1 year
Liu et al. (28)	USA	PCS	2016.05– 2018.04	DBD/ DCD	NMP	Self–developed device	21	84	35.0 ± 12.7	34.8 ± 15.0	57.0 ± 7.1	57.4 ± 8.4	19.1 ± 7.7	19.4 ± 8.7	3.2 ± 0.8	NA	20.75 ± 4.74^*^	N/A	1 year

*MCT, multicenter-clinical trials; N/A, non-available; RCT, randomized control trial; RCS, retrospective cohort study*;

* *, Functional warm ischemic time applies only to DCD donors*;

** *, In Watson's study, ECD livers(declined marginal livers) were used after NMP repair. &, MP group is significant higher compared with CS group; #, the total preservation time in this study*.

**Table 2:** Main outcomes of the included studies.

**References**	**Peak ALT**		**Peak AST**		**EAD**			**PNF**			**Total biliary complications**		
	**MP**	**CS**	**MP**	**CS**	**MP(%)**	**CS(%)**	**p**	**MP(%)**	**CS(%)**	**p**	**MP(%)**	**CS(%)**	***p***
Guarrera et al. (10)	560.0 ± 355.5 IU/mL	1358 ± 1208.4 IU/mL^*^	1154 ± 355.5 IU/mL	3339 ± 3376.9 IU/mL^*^	5	25	0.08	0	0	ns	10	20	N/A
Dutkowski et al. (22)	1239 (689–2126) U/L	2065 (1331–3596) U/L ^*^	1808 (1133–3547) U/L	2848 (1485–6724) U/L ^*^	20	44^*^	0.03	0	6	ns	20	46	0.035^*^
Guarrera et al. (21)	550 IU/mL	900 IU/mL #^*^	1300 IU/mL	1600 IU/mL #	19	30	0.384	3.2	6.7	0.612	13	43.3	0.001^*^
Ravikumar et al. (25)	N/A	N/A	417 (84–4681) U/L	902 (218–8786) U/L^*^	15	22.5	0.734	0	0	ns	N/A	N/A	N/A
Selzner et al. (23)	619 (55–2858) U/L	949 (233–3073) U/L	1182 (167–6700) U/L	1474 (521–5156) U/L	0	0	ns	0	0	ns	N/A	N/A	N/A
Bral et al. (24)	N/A	N/A	1252 (383–2600) U/L	839 (153–2600) U/L	55.6	29.6	0.23	0	0	ns	0	14.8	0.55
van Rijn et al. (26)	966 U/L	1858 U/L^*^	N/A	N/A	0	10	1.00	0	0	ns	40	55	N/A
Watson et al. (27)	1069 (187–4991) IU/L	787 (155–2238) IU/L	N/A	N/A	NA	NA	N/A	NA	NA	N/A	NA	NA	N/A
Nasralla et al. (18)	N/A	N/A	488.1 (408.9–582.8) U/L	964.9 (794.5–1,172.0) U/L^*^	10.1	29.9^*^	0.0002	0.8	0	ns	N/A	N/A	N/A
Ghinolfi et al. (30)	332 (263–610) U/L	428 (303–616) U/L	709.5 (371–1575) U/L	574 (377–1162) U/L	20.0	10.0	1.000	0	0	ns	10.0	0	1.000
Patrono et al. (29)	792 ± 773 U/L	817 ± 540 U/L	1425 ± 1729 U/L	1498 ± 1034 U/L	32	34	1.0	0	0	ns	24	18	0.76
Liu et al. (28)	1357 ± 1492 U/L	2615 ± 2541 U/L^*^	363 ± 318 U/L	1021 ± 999 U/L^*^	19	46.4	0.02	0	0	ns	N/A	N/A	N/A

*ns, not significant; N/A, non-available*;

* *, statistically significant; #, approximate number because only diagram form was available in this included study*.

### Meta-Analysis of Primary Outcomes

#### Liver Graft Injury

**Figure 2:**
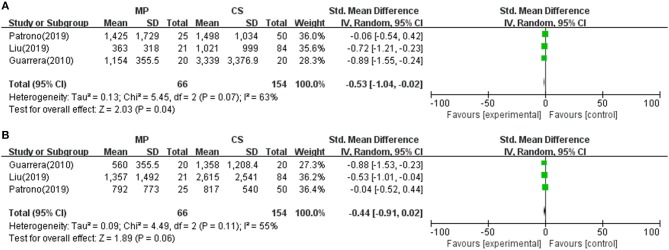
Meta-analysis on posttransplant peak AST/ALT levels between MP and CS preservation. **(A)** AST level. MP reduced posttransplant peak AST level (SMD = −0.53; 95% CI = −1.04 to −0.02; *P* = 0.04), **(B)** ALT level. MP tends to reduce posttransplant peak AST level (SMD = −0.44; 95% CI = −0.91 to −0.02; *P* = 0.06). MP, machine perfusion; CS, cold storage; SMD, standardized mean difference; CI, confidence interval.

Reperfusion injury and liver graft function were determined by AST and/or ALT serum levels after transplantation. As shown in Table 2, all 12 included studies reported posttransplant peak ALT and/or AST level within 1 week. Seven of the 12 included studies found a significantly lower posttransplant peak ALT and/or AST level during the first 7 days after surgery in the MP group compared with the CS group (*P* < 0.05) (10, 18, 21, 22, 25, 26, 28), whereas the rest (five studies) observed comparable hepatic transaminases levels between two groups (23, 24, 27, 29, 30). However, different studies reported transaminase by different data types and at variable time points. Only three studies reported posttransplant peak ALT and AST level by mean and standard deviation (10, 28, 29). Meta-analysis demonstrated that livers preserved with MP had significantly lower peak AST levels than CS (SMD = −0.53; 95% CI = −1.04 to −0.02; *P* = 0.04) (Figure 2). There was also a trend favoring the use of MP in reducing posttransplant peak ALT levels compared with CS (SMD = −0.44; 95% CI = −0.91 to −0.02; *P* = 0.06) (Figure 2).

Moreover, eight of the 12 included studies clinically measured the longitudinal serum hepatic transaminases levels in 1 or 2 weeks after transplantation. Except Bral and colleagues's trial (24), seven studies reported a faster decline in posttransplant hepatic transaminases levels in MP preservation recipients when compared to CS preservation recipients (10, 18, 21–23, 26, 30). Notably, six studies, including the large sample multicenter RCT by Nasralla et al. (18) and the RCT by Ghinolfi et al. (30), found statistically different reducing trends on posttransplant hepatic transaminases between the two groups within the first week after transplantation (*P* < 0.05) (10, 18, 21, 22, 26, 30).

### Early Allograft Dysfunction

**Figure 3:**
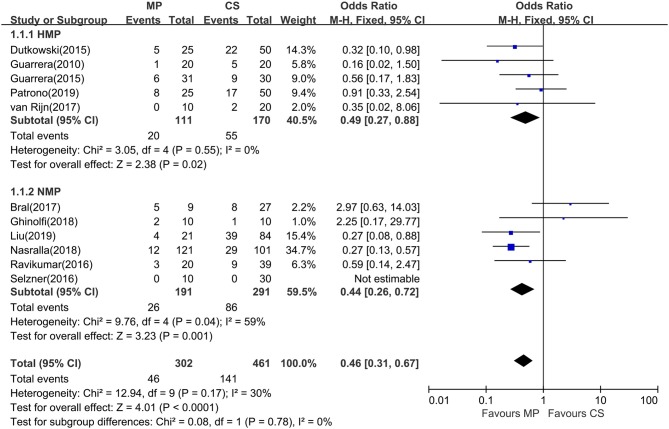
Meta-analysis on EAD rates between MP and CS preservation. HMP reduced the EAD rates (OR = 0.49; 95% CI = 0.27–0.88; *P* = 0.02), and NMP reduced the EAD rates (OR = 0.34; 95% CI = 0.20–0.59; *P* = 0.0001). EAD, early allograft dysfunction; MP, machine perfusion; CS, cold storage; HMP, hypothermic machine perfusion; NMP, normothermic machine perfusion; CI, confidence interval.

Eleven of the 12 included studies reported the incidence of EAD within the first week posttransplantation. The overall rate of EAD was 15.2% (range, 0–55.6%) for the MP group and 30.6% (0–46.4%) for the CS group. Pooled meta-analysis of the HMP and NMP studies revealed that the overall rate of EAD was significantly reduced in the MP preservation compared with the CS preservation (fixed effects: OR = 0.46; 95% CI = 0.31–0.67; *P* < 0.0001) (Figure 3). There was no statistically significant heterogeneity among the 11 included studies (χ^2^ = 12.94, *P* = 0.17, *I*^2^ = 30%).

Subgroup analysis showed no significant difference between two subgroups (*I*^2^ = 0%; *P* = 0.78). In the subgroup analysis, EAD rates were significantly lower in the HMP subgroup (fixed effects: OR = 0.49; 95% CI = 0.27–0.88; *P* = 0.02) compared to those of CS. There is statistically significant heterogeneity in the NMP subgroup (χ^2^ = 9.76, *P* = 0.04, *I*^2^ = 59%). Thus, a random-effects model was adopted. However, subgroup analysis showed no significant difference between NMP preservation and CS preservation (random effects: OR = 0.60; 95% CI = 0.24–1.52; *P* = 0.28, figure not shown). In the NMP subgroup, a sensitivity analysis was conducted by excluding Bral and colleagues' study (24) because of its heterogeneity in sample size and donor type; the risk of EAD reached statistical significance between two groups (fixed effects: OR = 0.34; 95% CI = 0.20–0.59; *P* = 0.0001). The overall risk of EAD in the MP group remained statistically significant (fixed effects: OR = 0.40; 95% CI = 0.27–0.60; *P* < 0.00001; χ^2^ = 7.07, *P* = 0.53, *I*^2^ = 0%) (figure not shown).

### Primary Nonfunction

**Figure 4:**
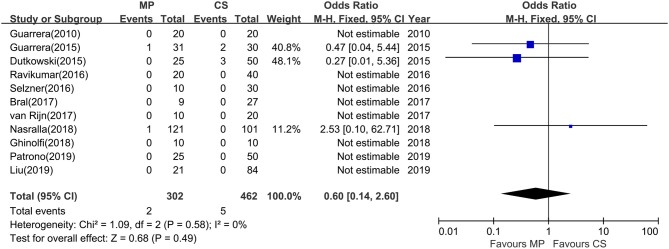
Meta-analysis on PNF rates between MP and CS preservation. MP cannot reduce the PNF rates (OR = 0.60; 95% CI = 0.14–2.60; *P* = 0.49). Data are expressed as OR ± 95% CI. PNF, primary nonfunction; MP, machine perfusion; CS, cold storage; CI, confidence interval.

Except Watson and colleagues' trial (27), the rest (11 studies) evaluated the rate of PNF after transplantation. However, episodes of PNF were observed in only three studies. The incidence of PNF was 0.7% (range, 0–3.2%) in the MP group and 1.1% (range, 0–6.7%) in the CS group, respectively. No heterogeneity was observed (χ^2^ = 1.09, *P* = 0.58; *I*^2^ = 0%); thus, a fixed-effects model was used. A meta-analysis indicated that there was no statistical significance in PNF rates between MP preservation and CS preservation (OR = 0.60; 95% CI = 0.14–2.60; *P* = 0.49) (Figure 4).

### Total Biliary Complications and Ischemic Cholangiopathy

**Figure 5:**
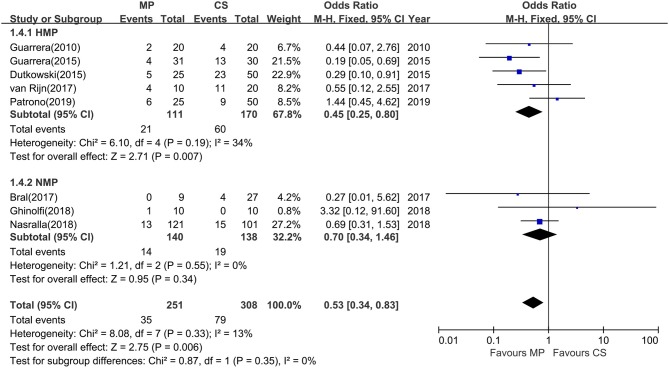
Meta-analysis on total biliary complications rates between MP and CS preservation. HMP reduced the total biliary complications (OR = 0.45; 95% CI = 0.25–0.80; *P* = 0.007), but NMP cannot reduce the total biliary complications (OR = 0.70; 95% CI = 0.34–1.46; *P* = 0.34). MP, machine perfusion; CS, cold storage; HMP, hypothermic machine perfusion; NMP, normothermic machine perfusion; CI, confidence interval.

All 12 studies reported biliary complications after transplantation within 1-year follow-up. However, two studies did not report detailed information of biliary complications (23, 28), and one study did not provide the complication data in the CS group (25), whereas Watson et al. trial reported only IC instead of all biliary complications. The rest (eight studies) compared the rates of total biliary complications between the MP and CS groups at 1 year after transplantation (Table 2). There was no heterogeneity among the eight studies (χ^2^ = 8.08, *P* = 0.33, *I*^2^ = 13%); thus, a fixed model was adopted. Meta-analysis of these studies indicated that total biliary complications at 1 year after transplantation were significantly lower in MP preservation group compared with CS preservation group (fixed-effects model: OR = 0.53; 95% CI = 0.34–0.83; *P* = 0.006) (Figure 5). Subgroup analysis showed that incidence of total biliary complications was significantly lower in HMP patients compared with CS patients (fixed-effects model: OR = 0.45; 95% CI = 0.25–0.80; *P* = 0.007). However, no significant difference could be detected between the NMP subgroup and CS subgroup (fixed-effects model: OR = 0.70; 95% CI = 0.34–1.46; *P* = 0.34) (Figure 5).

**Figure 6:**
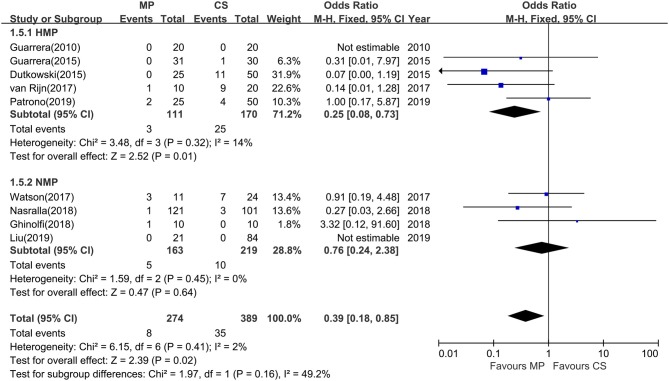
Meta-analysis on ischemic cholangiopathy rates between MP and CS preservation. HMP reduced the ischemic cholangiopathy rates (OR = 0.25; 95% CI = 0.08–0.73; *P* = 0.01), and NMP cannot reduce the ischemic cholangiopathy rates (OR = 0.76; 95% CI = 0.24–2.38; *P* = 0.64). Data are expressed as OR ± 95% CI. MP, machine perfusion; CS, cold storage; HMP, hypothermic machine perfusion; NMP, normothermic machine perfusion; CI, confidence interval.

Among the 12 included studies, nine studies further reported the incidence of IC at 1-year follow-up. The overall rates of IC were significantly lower in MP preservation patients compared with CS preservation patients (fixed-effects model: OR = 0.39; 95% CI = 0.18–0.85; *P* = 0.02). No significant heterogeneity was found between the MP and CS groups (χ^2^ = 6.15, *P* = 0.41; *I*^2^ = 2%). The subgroup analysis showed that the incidence of IC was significantly lower in HMP preservation patients compared with CS preservation patients (OR = 0.25; 95% CI = 0.08–0.73; *P* = 0.01) (Figure 6), but there is no statistical difference in IC rates between NMP preservation and CS preservation (OR = 0.76; 95% CI = 0.24–2.38; *P* = 0.64) (Figure 6).

### Hepatic Artery Thrombosis

Seven studies, including four HMP studies and three NMP studies, compared the risk of posttransplant HAT between the two preservation technologies. The overall incidence of HAT was 2.1% (range, 0–10.0%) in the MP group and 5.2% (range, 0–10.0%) in the CS group, respectively. Hepatic artery thrombosis rates were lower in both the HMP group (fixed-effects analysis: OR = 0.46, 95% CI = 0.12–1.77; *P* = 0.26) and NMP group (fixed-effects analysis: OR = 0.67; 95% CI = 0.16–2.77; *P* = 0.58) compared to those of the CS group. However, there was no statistical significance (*P* > 0.05). A pooled meta-analysis of HMP and NMP studies indicated that there was no statistical significance in HAT rates between MP preservation and CS preservation (OR = 0.55; 95% CI = 0.21–1.44; *P* = 0.22) (Figure S1).

### Postreperfusion Syndrome

Four studies reported the rates of PRS. Postreperfusion syndrome rates were lower in the MP preservation group compared with the CS group (random-effects effect: OR = 0.70; 95% CI = 0.18–2.73; *P* = 0.61). However, it had no statistical significance (Figure S2).

### Patient Survival and Liver Graft Survival

To evaluate the posttransplant survival outcomes of MP preservation vs. CS preservation, the data of graft survival and patient survival at 1 month, 6 month, and 1 year were extracted and analyzed (Table S2). The meta-analysis indicated that there was no difference in graft survival between MP preservation patients and CS preservation at 1 month (seven studies supported, OR = 0.78; 95% CI = 0.21–2.88; *P* = 0.72; χ^2^ = 3.54, *P* = 0.32; *I*^2^ = 15%, figure not shown), 6 months (six studies supported, OR = 0.80; 95% CI = 0.26–2.46; *P* = 0.69; χ^2^ = 5.23, *P* = 0.26; *I*^2^ =23%, figure not shown), and 1 year (eight studies supported, OR = 1.15; 95% CI = 0.59–2.22; *P* = 0.68; χ^2^ = 6.00, *P* = 0.42; *I*^2^ = 0%, Figure S3).

As for patient survival (Table S3), the meta-analysis indicated that there was no difference in patient survival between MP preservation patients and CS preservation patients at 1 month (seven studies supported, OR = 0.37; 95% CI = 0.07–1.92; *P* = 0.24; χ^2^ = 1.61, *P* = 0.45; *I*^2^ = 0%, figure not shown), 6 months (six studies supported, OR = 0.58; 95% CI = 0.19–1.74; *P* = 0.33; χ^2^ = 4.30, *P* = 0.51; *I*^2^ =0%, figure not shown), and 1 year (eight studies supported, OR =1.10; 95% CI = 0.55–2.19; *P* = 0.79; χ^2^ = 5.04, P = 0.65; *I*^2^ =0%, Figure S4).

### Publication Bias

Publication bias is formally determined through funnel plotting effect sizes. No evidence of publication bias was found in the comparison of EAD, PNF, total biliary complications, IC, and 1-year graft/patient survival analysis (data not shown).

## Discussion

The results of this meta-analysis provide a comprehensive and up-to-date insight into the current evidence regarding the priority of MP preservation to CS in the clinical setting. Our analysis concludes that compared with CS preservation MP can reduce the risk of post-LT EAD and total biliary complications. However, MP preservation could not improve PNF, 1-year graft, and patient survival.

Theoretically, MP preservation can simulate physiological condition *ex vivo* after donor liver procurement by effectively providing continuous circulation of oxygen, essential nutrients, and adequate adenosine triphosphate substrates to restore the normal physiological and also supplying vasodilators to maintain the peribiliary vascular microcirculation while flushing out the metabolites to avoid accumulation of toxic substances. Evidence from animal models and human studies suggests that MP can avoid cold ischemia injury during preservation and reduce posttransplant IRI and sterile inflammation, thus causing less damage to hepatocytes and the biliary epithelia in both deceased brain donor (DBD) and DCD LT compared with CS preservation. As a result, MP has been found to bring about significantly reduced posttransplant peak enzyme release, lower EAD rates, less biliary complications, and shorter hospital stay in MP livers.

Liver graft reperfusion injury was usually determined by AST and/or ALT serum levels after transplantation. Previous evidence suggested that episodes of EAD and elevation of hepatic transaminases may adversely affect survival rates after LT (17). Although the present analysis for posttransplant AST/ALT comparisons was limited by different data types and variable time points during recording clinical data in different studies, our systematic review did show that all 12 included studies observed posttransplant AST/ALT reductions in the MP preservation group, with seven studies reaching statistical significance (Table 2). Further pooled meta-analysis did confirm the use of MP in reducing posttransplant peak AST levels (*P* < 0.05), and there was a trend favoring MP in ALT levels (*P* = 0.06) (Figure 2). Notably, 10 studies among all included studies showed lower EAD rates in the MP group, with pooled meta-analysis reaching statistical significance (Figure 3, *P* < 0.0001). In line with animal studies, our meta-analysis suggested that MP can achieve definite protecting effects for reducing IRI by decreasing transaminases releasing.

As expected, a definite reduction in the overall risk of EAD was confirmed in MP preservation recipients when compared with CS preservation (OR = 0.46; *P* < 0.0001) (Figure 3), indicating MP's excellent protective effects in hepatocellular function. It is notable that these reductions in peak hepatic transaminases and EAD rates were achieved in the context of using DCDs or ECDs with longer functional warm ischemia times (WITs) and longer preservation times in the MP group (Table 1), suggesting that MP may have substantial benefits in the desired objective of expanding donor pool and increasing organ utilization without compromising outcome.

Biliary complications have shown to be the most frequent complications after DCD LT. Nonanastomotic biliary strictures or IC is one of the most feared complications of DCD LT. Specially, DCD recipients have a threefold higher risk of developing nonanastomotic biliary strictures after transplantation compared with DBD recipients (31). Long periods of donor warm ischemia in DCD donation are believed to be responsible for IC and graft loss. One important benefit of MP may be its ability to maintain the peribiliary vascular microcirculation and restore the normal physiological environment of biliary tree, which has been shown to reduce biliary IRI and biliary complications. The principal finding in this study is that MP preservation can significantly reduce total biliary complications at 1 year after transplantation (OR = 0.53; *P* = 0.006) (Figure 5). Furthermore, the rates of IC were significantly lower in the MP group compared with the CS group (OR = 0.39; *P* = 0.02) (Figure 6). Van Rijn et al. (6) further investigated the protective effect of MP on histopathological level in their trial, showing that MP significantly reduced the injury of the deep peribiliary glands and the degree of stroma necrosis. Machine perfusion can resuscitate the DCDs from long functional warm ischemia and further protect the vulnerability of the biliary tree from IRI, which may substantially reduce biliary complications, expand the donor poor, and increase DCD utilization.

In the current analysis, no significant effects could be detected favoring the use of MP in reducing PNF rates compared with CS (OR = 0.60; *P* = 0.49). This may be true, or more likely, there may be insufficient data to draw the conclusions about the benefits of MP on reducing PNF rates as clinical investigations on MP in LT are still limited. Primary nonfunction rates are relatively low in clinical practice, occurring in 5% to 8% of liver transplants, but it may be a life-threatening circumstance requiring immediate retransplantation (32, 33). In this study, the episodes of PNF occurred in only one HMP-ECD liver in Guarrera and colleagues' study, one NMP liver in Nasralla and colleagues' study, and five CS preservation livers in two studies, whereas the other studies reported no incidence of PNF in either MP or CS preservation groups (Table 2). The highest incidence of PNF remained only 6.7% in CS-ECD in Guarrera and colleagues' study (21). Thus, no difference seen in terms of reducing PNF rates may be due to an inability to detect an effect of MP because of the very low incidence of PNF after transplantation. Further large sample clinical studies are needed to clarify this point.

Our present meta-analysis identifies no significant difference in either 1-month, 6-month, or 1-year graft and patient survival between MP preservation recipients and CS preservation recipients (Figures S3, S4). Actually, 11 of the 12 included studies reported no significant improvement in graft and patient survival after MP preservation. Only in Dutkowski et al. (22) retrospective comparative analysis, 1-year graft survival was significantly improved in the MP-DCD group compared to the CS-DCD group (*P* = 0.035). Although there was a trend favoring MP preservation in Patrono and colleagues' study (29), with graft survival rates within the first year almost reaching significant level (*P* = 0.18), a longer follow-up result was lacking. In Dutkowski and colleagues' recent serial study (34), they first reported 5-year graft survival after censoring tumor recurrence was significantly improved by HOPE, reaching 94% in the HOPE-treated DCD group, compared to 78% in the untreated DCD group (*n* = 50, *P* = 0.024) (Figure 3), suggesting the superiority of MP preservation in DCD livers. However, this small-number cohort study has limitations such as retrospective design, different implantation techniques, and inconsistent immune suppression between the MP and CS groups, highlighting the necessity for large-sample RCTs. In pig models, the recent meta-analysis identified no significant difference in 5- to 7-day survival rate between the MP group and CS group (9). Therefore, no clear evidence yet can be drawn to conclude MP's superiority to CS preservation in posttransplant survival within 1-year follow-up. Definite superiority of MP preservation in graft and patient survival compared with CS preservation still needs to be investigated in future RCTs with longer follow-up.

By reducing IRI and offering the opportunity for *ex vivo* graft repair through delivery of targeted additives, MP seems to provide more obviously protective effects in relatively low-quality donors such as ECDs or DCDs. Thus, MP has the potential to resuscitate the donor graft from IRI to a better state, reduce the discarded rates, and increase the overall organ utilization. The results of this study on MP's benefits are consistent with those findings in kidney transplantation. In kidney transplantation, large RCTs and recent meta-analyses have drawn the conclusions of MP's superiority to CS on improvement in short-term outcomes, such as enhanced early renal function and decreased risk of DGF in DCD and ECD grafts. Similar to our analysis, however, no significant differences could be found in PNF rates and patient survival between MP and CS for DCD LT (35). In one recent meta-analysis of NMP-assisted pig LT (12), both *ex vivo* and *in situ* NMP could significantly reduced peak AST and ALT levels compared with CS for DCD livers (*P* < 0.00001). It is worth noting that total bile production was also significantly higher (mean = 174 mL; CI, 155–193; *P* < 0.0001) in the NMP group, indicating the excellent effect of NMP in preserving liver functions and viability assessment. In Zhang and colleagues' recent meta-analysis on HMP, they found that HMP could significantly reduce the incidences of EAD (OR = 0.36; 95% CI = 0.17–0.77; *P* = 0.008) and biliary complications (OR = 0.47; 95% CI = 0.28–0.76; *P* = 0.003), respectively. And there was no difference in the incidence of PNF (OR = 0.30; 95% CI = 0.06–1.47; *P* = 0.14) and vascular complications (OR = 0.69; 95% CI = 0.29–1.6; *P* = 0.41) between HMP and CS preservation. These results were consistent with our present analysis. However, Zhang et al. found that 1-year graft survival was significantly increased in HMP preservation compared to CS preservation (OR = 2.19; 95% CI = 1.14–4.20; *P* = 0.02), in which there was no difference in our analysis (OR = 1.15; 95% CI = 0.59–2.22; *P* = 0.42). To be noted, Zhang and colleagues' meta-analysis included overlapping studies from the same institution [Guarrera et al. (21) and Schlegel et al. (34)], which may magnify the protective effects of HMP on outcomes. Moreover, the included study of Dutkowski et al. (22) in Zhang and colleagues' meta-analysis did not control the donor type, with DCD livers in the HMP group and DBD livers in the CS group, which may bring some bias in the meta-analysis. Therefore, the benefits of MP on long-term survival required further researches. Combining aforementioned findings in kidney transplantation, MP in pig LT, and the results of this study, it is reasonable to conclude that MP preservation could enhance early graft function recovery and improve short-term outcomes after LT. However, there may be less-clear benefits in the longer-term graft and patient outcomes. In Tchilikidi's (36) systematic review, the short-term outcomes and 30-day mortality had no difference between NMP and CS by comprehensive analysis on large literatures. The protective effect of MP needs to be validated in more large multicenter prospective trials with larger samples and longer-term follow-up. In recent years, a batch of human clinical trials involving the use of HMP or NMP in LT was implemented to further validate MP's safety, feasibility, and its superiority on long-term outcomes (36). The results of some important RCTs are coming out in the next few years, which may greatly propel the development of MP's clinical applications (2, 36). Future meta-analyses are necessary to confirm the recommendation of MP in human LT.

The present study has several limitations. First, clinical heterogeneity between the included studies might still exist, although we had adopted strict enrollment criteria during screening the references. Selection bias due to different donor types, perfusion models, perfusion routes, hemodynamics, cold ischemia times (CITs), and perioperative parameter among different publications may still result in analytical bias. Notably, a recent meta-analysis did not observe any differences in post-LT outcomes such as PNF, biliary complication, and 1-year survival while using different perfusion fluids, volumes, or perfusion routes (37), indicating these factors may have less effect on the LT outcomes. Second, the number and the sample size of included studies are relatively small as clinical application of MP in LT and the completed human cases are still limited. There is unlikely to be adequate power to identify differences in relatively low-risk complications such as PNF. Also, the follow-up in the majority of included studies is relatively short, and very few studies reported 1-year graft and patient survival. Thus, no sufficient evidence can be obtained to find differences in graft and patient survival. In addition, this study presented data only about the short-term patient and graft survival, the influence of MP on long-term survival remains unclear. Third, the MP technique used in clinical setting is still under the stages of preliminary clinical trial. The meta-analysis data in this study mainly came from small-number trials, and the overall level of clinical evidence is not high. Considering these limitations in study methodology and sample size, further large-sample RCTs are needed to further confirm the benefits of MP on the prognosis of LT.

In conclusion, this meta-analysis demonstrates that MP preservation, especially HMP, could significantly improve short-term outcomes by reducing the rates of EAD, total biliary complications, and IC after transplantation compared to CS preservation. However, it was not correlated with the incidence of PNF, HAT, and 1-year graft and patient survivals. Considering the benefits, MP preservation is still recommended for LT to expand the donor pool and improve posttransplant outcomes.

## Data Availability Statement

The raw data supporting the conclusions of this article will be made available by the authors, without undue reservation, to any qualified researcher

## Author Contributions

JJ and YN designed the research. JJ, YN, JL, and HX retrieved literature, extraction data, analyzed the data, and wrote the manuscript. JL, HX, LZ, JY, and S-SZ revised the manuscript.

### Conflict of Interest

The authors declare that the research was conducted in the absence of any commercial or financial relationships that could be construed as a potential conflict of interest.

## Abbreviations

AST: aspartate aminotransferase
ALT: alanine transaminase
CI: confidence interval
CS: cold storage
DBD: deceased brain donor
DCD: donor after cardiac death
EAD: early allograft dysfunction
ECD: extended criteria donors
HAT: hepatic artery thrombosis
HMP: hypothermic machine perfusion
HOPE: hypothermic oxygenated perfusion
IRI: ischemia–reperfusion injury
IC: ischemic cholangiopathy
LT: liver transplantation
MP: machine perfusion
MELD: model for end-stage liver disease
NOS: Newcastle–Ottawa Quality Assessment Scale
NMP: normothermic machine perfusion
OR:odds ratio
PNF: primary nonfunction
PRISMA: Preferred Reporting Items for Systemic Reviews and Meta-Analysis.
