# Supplementary material for: A Systematic Review and Meta-Analysis of Machine Perfusion vs. Static Cold Storage of Liver Allografts on Liver Transplantation Outcomes: The Future Direction of Graft Preservation
Source: Front Med (Lausanne). 2020 May 12;7:135. doi: 10.3389/fmed.2020.00135 (PMC7247831; doi:10.3389/fmed.2020.00135)
Supplement: Supplementary file 1 [file Data_Sheet_1.docx]

**Supplementary tables and figures:**

**Supplementary Table Legends**

**Table S1 Quality ratings for the 8 included cohort studies on the basis of Newcastle-Ottawa quality assessment scale**

| **Quality assessment of the included non- randomized clinical trials** | | | | | | | | | |
| --- | --- | --- | --- | --- | --- | --- | --- | --- | --- |
| **Reference** | **Selection** |  |  |  | **Comparability** | **Outcome** |  |  | **Total Score** |
|  | Representative of exposed cohort | Selections of non exposed cohort | Ascertainment of exposure | Absence of outcome at start of study | Control for donor and recipient age, MELD score, donor risk index, warm ischemia time | Assessment of outcome | Enough follow-up period(1 year ) | Adequacy of  follow-up |  |
| **Guarrera et al. (2010)** | 1 | 1 | 1 | 1 | 1 | 1 | 1 | 1 | 8 (high) |
| **Dutkowski et al. (2015)** | 1 | 0 | 1 | 1 | 2 | 1 | 1 | 1 | 8 (high) |
| **Guarrera et al. (2015)** | 1 | 1 | 1 | 1 | 1 | 1 | 1 | 1 | 8 (high) |
| **Ravikumar st al. (2016)** | 1 | 1 | 1 | 1 | 1 | 1 | 0 | 1 | 7 (fair) |
| **Selzner et al. (2016)** | 1 | 1 | 1 | 1 | 1 | 1 | 0 | 1 | 7 (fair) |
| **Bral et al. (2017)** | 1 | 1 | 1 | 1 | 2 | 1 | 0 | 1 | 8 (high) |
| **van Rijn et al. (2017)** | 1 | 1 | 1 | 1 | 1 | 1 | 1 | 1 | 8 (high) |
| **Watson et al. (2017)** | 0 | 1 | 1 | 1 | 1 | 1 | 1 | 1 | 7 (fair) |
| **Patrono et al. (2019)** | 1 | 1 | 1 | 1 | 2 | 1 | 1 | 1 | 9 (high) |
| **Liu Q et al. (2019)** | 1 | 1 | 1 | 1 | 2 | 1 | 1 | 1 | 9 (high) |
|  |  |  |  |  |  |  |  | Mean | 7.9 points |
| **Quality assessment of the bias risk for the included randomized clinical trial** | | | | | | | | | |
| **Reference** | Analysis strategy (intention to treat) | Jadad Score | Sequence generation | Allocation concealment | Blinding of participants | Blinding outcomes | Incomplete outcome data | Selective reporting | Other risk of bias |
| **Nasralla et al. (2018)** | Per protocol | 3 points | Low risk | Low risk | High risk | Low risk | Low risk | Low risk | Unclear |
| **Ghinolfi et al.(2018)** | Per protocol | 3 points | Low risk | Low risk | Low risk | Low risk | Low risk | Low risk | Unclear |

**Table S2 Graft survival of the included studies.**

| Reference | 1 month | | | 6 month | | | 1 year | | |
| --- | --- | --- | --- | --- | --- | --- | --- | --- | --- |
|  | MP(%) | CS(%) | p | MP(%) | CS(%) | p | MP(%) | CS(%) | p |
| Guarrera(2010) | 97.5 | 100 | ns | 90 | 97.5 | ns | 90 | 90 | ns |
| Dutkowski(2015) | N/A | N/A | -- | N/A | N/A | -- | 92 | 70 | 0.035 |
| Guarrera(2015) | N/A | N/A | -- | N/A | N/A | -- | 81 | 80 | 0.761 |
| Ravikumar(2016) | 100 | 97.5 | ns | 100 | 97.5 | 1 | 95 | N/A | -- |
| Selzner(2016) | 100 | 100 | ns | N/A | N/A | -- | N/A | N/A | -- |
| Bral(2017) | 100 | 100 | ns | 89 | 100 | 0.25 | N/A | N/A | -- |
| van Rijn(2017) | 100 | 85 | ns | 100 | 80 | ns | 100 | 70 | 0⋅052 |
| Watson(2017) | N/A | N/A | -- | N/A | N/A | -- | 83 | 88 | ns |
| Nasralla(2018) | N/A | N/A | -- | N/A | N/A | -- | 95 | 96 | 0.695 |
| Ghinolfi(2019) | 90 | 100 | ns | 90 | 90 | ns | 90 | 90 | ns |
| Patrono(2019) | 100 | N/A | ns | 100 | N/A | ns | 100 | 94 | ns |
| Liu Q(2019) | 95.2 | 100 | ns | 95.2 | 100 | ns | 95.2 | 100 | ns |

ns: not significant; N/A: non-available

**Table S3 Patient survival of the included studies.**

| Reference | 1 month | | | | 6 month | | | | | | 1 year | | | | |
| --- | --- | --- | --- | --- | --- | --- | --- | --- | --- | --- | --- | --- | --- | --- | --- |
|  | MP(%) | CS(%) | p | | MP(%) | | CS(%) | | p | | MP(%) | | CS(%) | | p |
| Guarrera(2010) | 97.5 | 100 | ns | | 90 | | 97.5 | | ns | | 90 | | 90 | | ns |
| Dutkowski(2015) | N/A | N/A | -- | | N/A | | N/A | | -- | | N/A | | N/A | | -- |
| Guarrera(2015) | N/A | N/A | -- | | N/A | | N/A | | -- | | 84 | | 80 | | 0.761 |
| Ravikumar(2016) | 100 | 97.5 | ns | | 100 | | 97.5 | | ns | | 95 | | N/A | | -- |
| Selzner(2016) | 100 | 100 | ns | | N/A | | N/A | | -- | | N/A | | N/A | | -- |
| Bral(2017) | 100 | 100 | ns | | 89 | | 100 | | 0.25 | | N/A | | N/A | | -- |
| van Rijn(2017) | 100 | 100 | ns | | 100 | | 97.5 | | ns | | 100 | | 85 | | 0⋅209 |
| Watson(2017) | N/A | N/A | -- | | N/A | | N/A | | -- | | 92 | | 96 | | ns |
| Nasralla(2018) | N/A | N/A | -- | | N/A | | N/A | | -- | | 94.9 | | 95.8 | | 0.901 |
| Ghinolfi(2019) | 100 | 100 | ns | 100 | | 90 | | ns | | 100 | | 90 | | ns | |
| Patrono(2019) | 100 | N/A | ns | | 100 | | N/A | | ns | | 100 | | 92 | | ns |
| Liu Q(2019) | 95.2 | 100 | ns | | 95.2 | | 100 | | ns | | 95.2 | | 100 | | ns |

ns: not significant; N/A: non-available

**Supplementary Figure Legends**

Figure S1. Meta-analysis on hepatic artery thrombosis rates between MP and CS preservation. MP = machine perfusion, CS = cold storage, HMP = hypothermic machine perfusion, NMP = normothermic machine perfusion, CI = confidence interval.

Figure S2. Meta-analysis on post-reperfusion syndrome rates between MP and CS preservation. MP = machine perfusion, CS = cold storage, CI = confidence interval.

Figure S3. Meta-analysis on one year graft survival between MP preservation and CS preservation. MP = machine perfusion, CS = cold storage, HMP = hypothermic machine perfusion, NMP = normothermic machine perfusion, CI = confidence interval.

Figure S4. Meta-analysis on one year patient survival between MP preservation and CS preservation. MP = machine perfusion, CS = cold storage, HMP = hypothermic machine perfusion, NMP = normothermic machine perfusion, CI = confidence interval.
