# Supplementary figures and images for: A Systematic Review and Meta-Analysis of Machine Perfusion vs. Static Cold Storage of Liver Allografts on Liver Transplantation Outcomes: The Future Direction of Graft Preservation
Source: Front Med (Lausanne). 2020 May 12;7:135. doi: 10.3389/fmed.2020.00135 (PMC7247831; doi:10.3389/fmed.2020.00135)

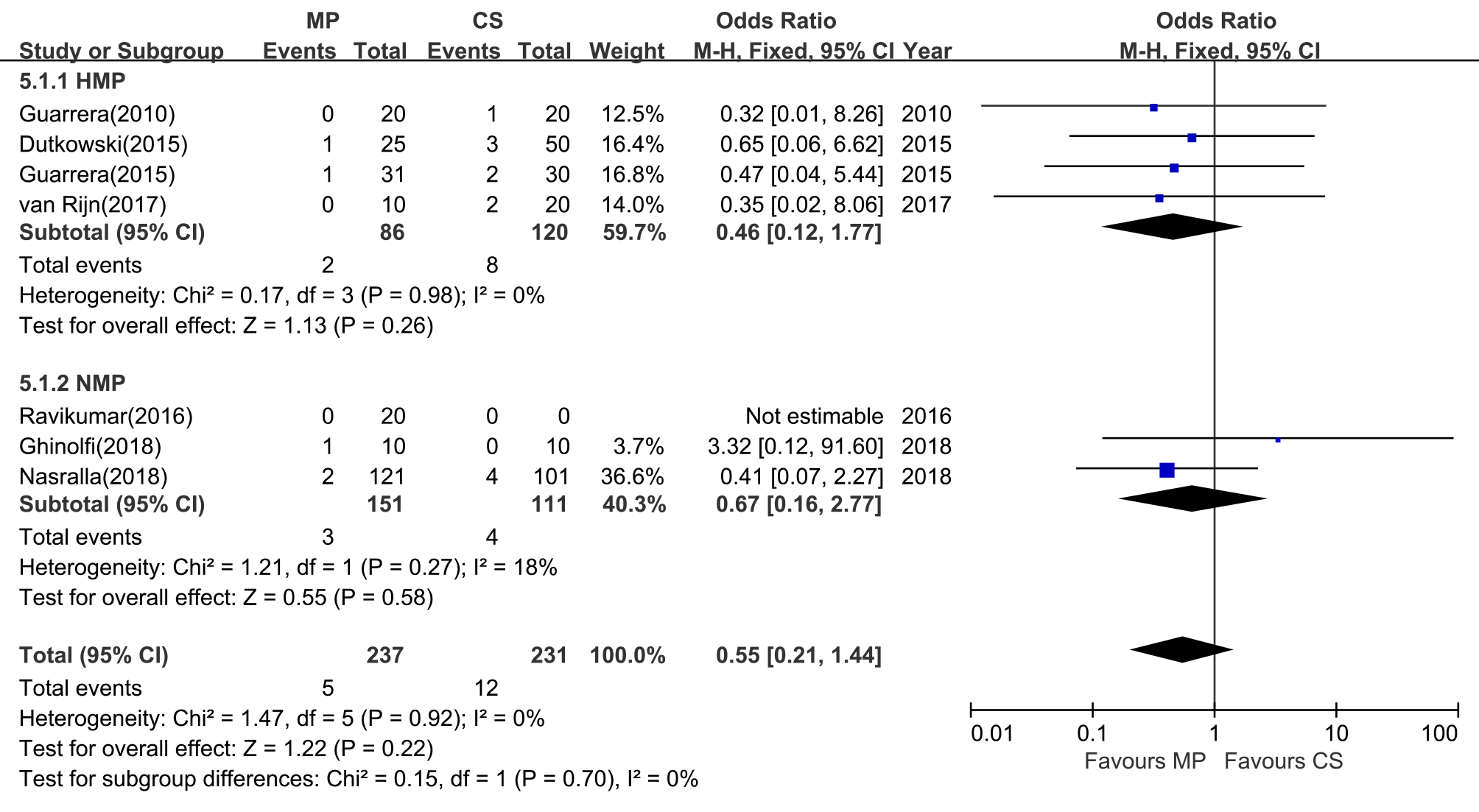

Supplement: Supplementary file 2 [file Image_1.tif]

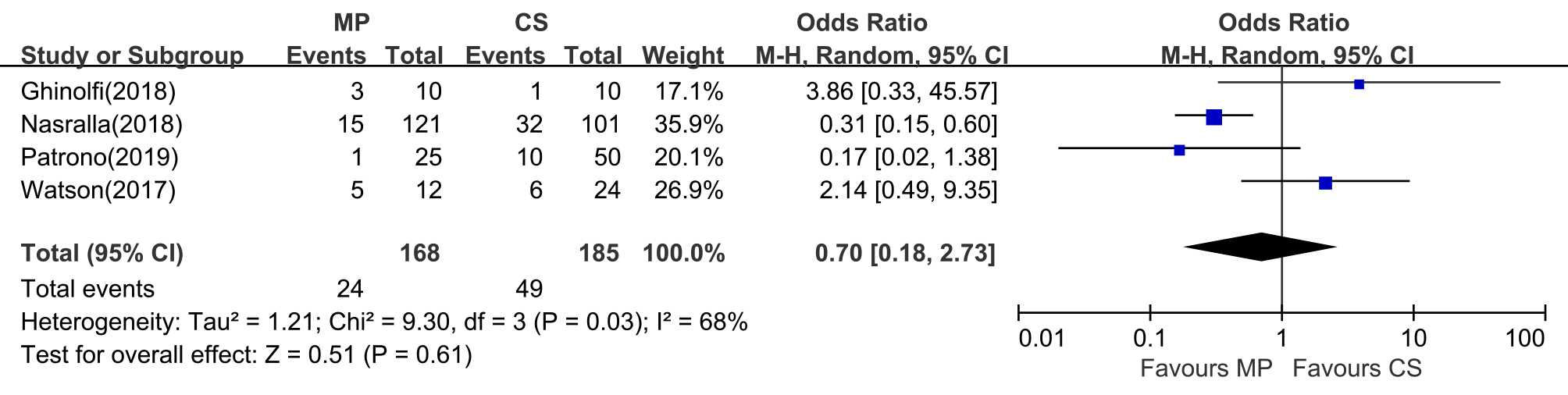

Supplement: Supplementary file 3 [file Image_2.tif]

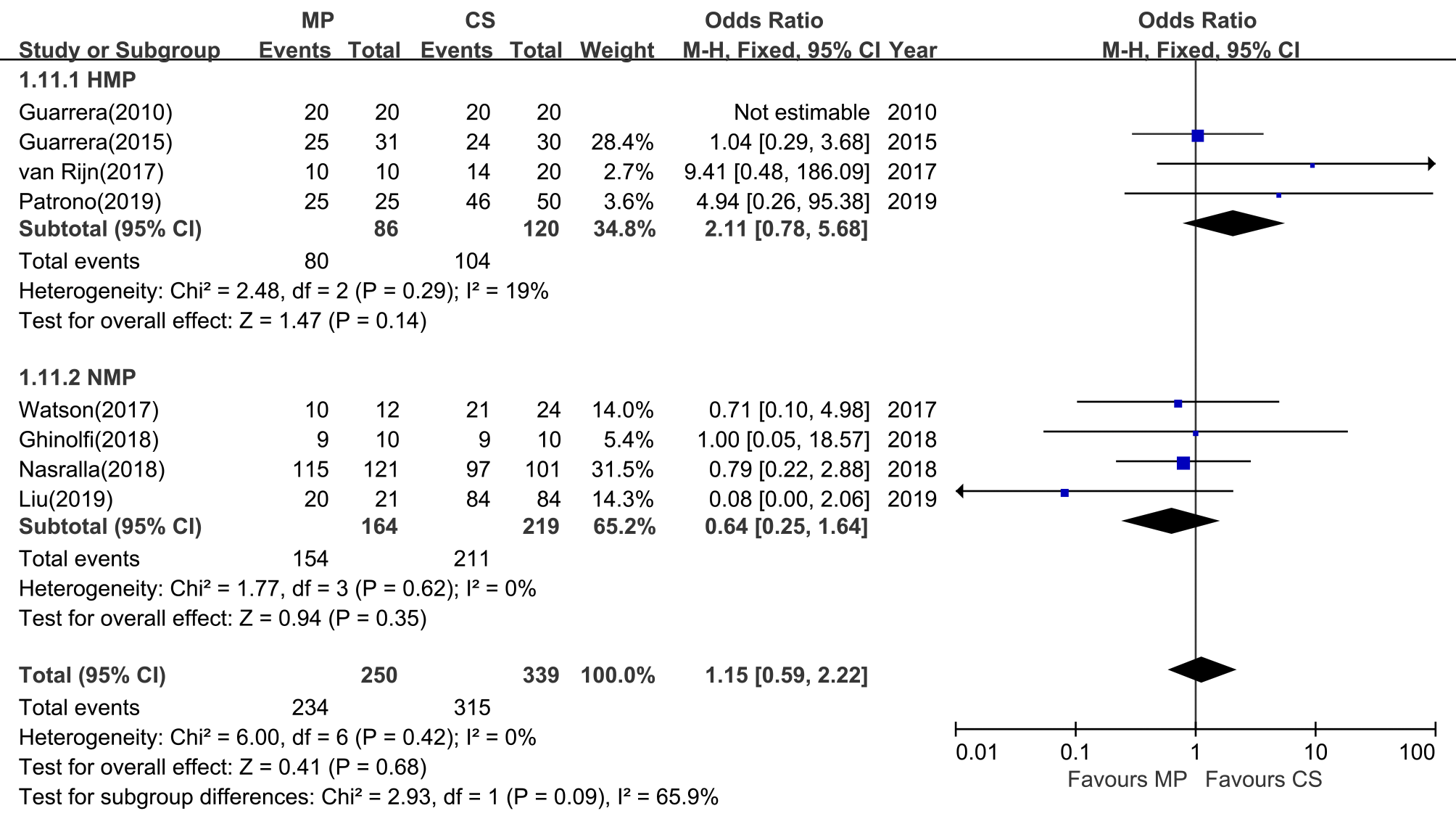

Supplement: Supplementary file 4 [file Image_3.tif]

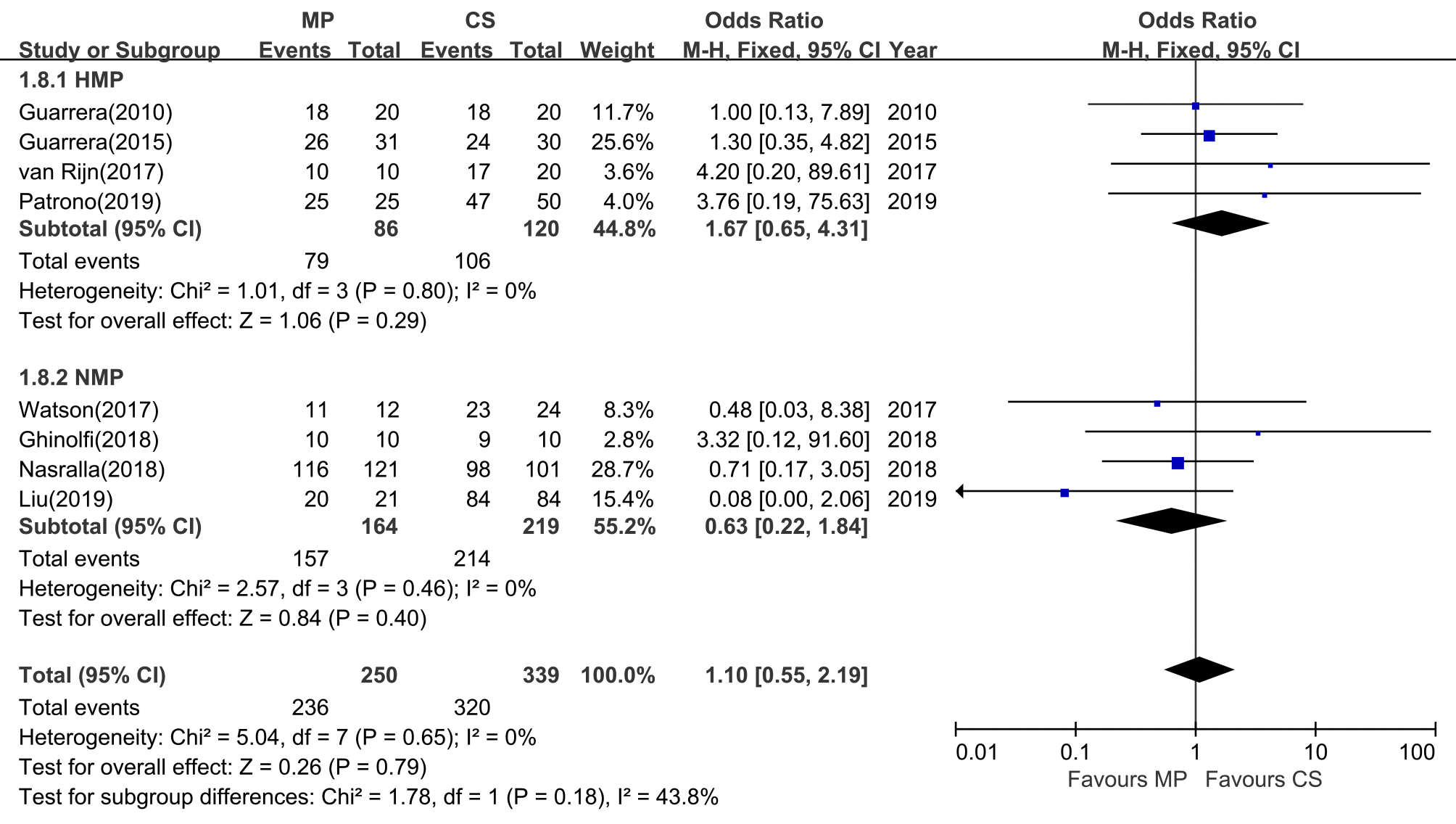

Supplement: Supplementary file 5 [file Image_4.tif]
